# Pharmacists’ role in diabetes management for persons with lived experience of homelessness in Canada: A qualitative study

**DOI:** 10.3389/fcdhc.2022.1087751

**Published:** 2022-12-22

**Authors:** Breanna McSweeney, Rachel B. Campbell, Eshleen K. Grewal, David J. T. Campbell

**Affiliations:** ^1^ Department of Medicine, Cumming School of Medicine, University of Calgary, Calgary, AB, Canada; ^2^ Department of Community Health Sciences, Cumming School of Medicine, University of Calgary, Calgary, AB, Canada; ^3^ Department of Cardiac Sciences, Cumming School of Medicine, University of Calgary, Calgary, AB, Canada

**Keywords:** diabetes, homelessness, pharmacists, pharmacy services, access to care

## Abstract

**Introduction:**

Persons with lived experience of homelessness face many challenges in managing diabetes, including purchasing and storing medications, procuring healthy food and accessing healthcare services. Prior studies have found that pharmacy-led interventions for diabetes improved A1C, and lowered blood pressure and cholesterol in general populations. This study evaluated how select pharmacists in Canada have tailored their practices to serve persons with lived experiences of homelessness with diabetes.

**Methods:**

We conducted a qualitative descriptive study using open-ended interviews with inner-city pharmacists in select Canadian municipalities (Calgary, Edmonton, Vancouver, and Ottawa). We used NVivo qualitative data analysis software to facilitate thematic analysis of the data, focusing on how pharmacists contributed to diabetes care for persons with lived experience of homelessness.

**Results:**

These pharmacists developed diabetes programs after discovering an unmet need in the population. Pharmacists have the unique ability to see patients frequently, allowing tailored education and hands-on assistance with diabetes management. These pharmacists provided extra-ordinary care like financial and housing resources and many of them were uniquely embedded within other services for persons with lived experience of homelessness (i.e. housing and social work supports). Pharmacists reported struggling with balancing optimal medical care for individuals with the financial constraints of running a business.

**Conclusion:**

Pharmacists are vital members of the diabetes care team for persons with lived experience of homelessness. Government policies should support and encourage unique models of care provided by pharmacists to improve diabetes management for this population.

## Introduction

Persons with lived experience of homelessness (PWLEH) have a higher mortality rate than housed individuals due to a variety of reasons including increased rates of mental health disorders and/or substance use, and poor access to health care and medications (1). Specifically, among those with diabetes, PWLEH in Canada have been shown to have a 45% increase in the risk of death, compared to those who have stable housing (2). PWLEH are known to have higher blood glucose and higher rates of diabetes-related complications, including hypoglycemia, hyperglycemic emergencies, and foot pathology (1, 3, 4). Barriers to diabetes self-management include a lack of healthy food options at shelters and safe medication (including insulin) storage (5, 6). PWLEH often have to decide between buying food or refilling prescription medication due to lack of medication coverage and/or medications being lost or stolen (6, 7).

Diabetes Canada Clinical Practice Guidelines recommend that pharmacists be involved in the multidisciplinary team to help manage all patients living with diabetes (8). Pharmacists’ roles can include patient education, medication reviews, and coordination with other care providers. Furthermore, in some Canadian jurisdictions, pharmacists have an expanded scope of practice that includes the ability to prescribe medications and order laboratory investigations (9). Studies have shown that when pharmacists are involved it can help those with diabetes to reach A1C, LDL cholesterol and blood pressure targets (10). The involvement of pharmacists in the care of PWLEH may be particularly important given the fact that many in this population lack primary care physicians (11). Pharmacists are often regarded as the most accessible primary care provider due to their locations in the community and the fact that appointments are not required to be seen in consultation (12). Bahrami et al. found that the addition of a pharmacist completing consults at a homeless shelter in Dallas, Texas significantly improved the glycemia of PWLEH (13). Information on how pharmacists design and tailor their practice to achieve these improved outcomes for PWLEH is limited.

Our objective was to describe the approaches of four pharmacists across Canada who work closely with PWLEH. A prior publication by our team discussed the general model of care (14) but here we discuss the details of how pharmacists can provide diabetes care for PWLEH.

## Methods

### Study design

We conducted a multi-site qualitative descriptive study using the constructivist paradigm. We interviewed providers from programs that were tailored to address the needs of people experiencing homelessness with diabetes in four large Canadian urban centres (Calgary, Edmonton, Vancouver and Ottawa). The interviews took place between July 2018 and February 2019. This study was approved by the Research Ethics Boards of the University of Calgary and Unity Health Toronto/St. Michael’s Hospital. We report our findings according to the Standards for Reporting Qualitative Research (SRQR – **Appendix A**).

### Sampling and Recruitment

We recruited participants who had at least 3 years of providing direct clinical care to those with diabetes and homelessness. We sought pharmacists who worked in inner-city settings serving PWLEH and who had an explicit focus or program for these populations. These pharmacists were recruited through internet searches and snowball sampling of health agencies serving PWLEH in these cities.

### Data collection

Data were collected using semi-structured in-person qualitative interviews, conducted by research personnel (RBC and DJTC) who are experienced in qualitative data collection and analysis, and who have expertise in homelessness and diabetes care for PWLEH, respectively. Pre-defined interview guides were created (**Appendix B**); these were iterative and after each interview, the study team members reconsidered the interview guide, adding additional questions to probe into areas that were revealed in prior interviews. Interviews ranged from 20-90 minutes in duration. Written informed consent was obtained from each participant. Interviews were digitally recorded, and recordings were subsequently transcribed verbatim by a trained professional.

### Data analysis

Data analysis was facilitated by NVivo 12 qualitative data analysis software (Doncaster, Australia). A thematic analysis of the transcripts with pharmacists was undertaken. The transcripts were initially coded by two independent analysts (RB and EG) for themes and codes (open coding). During the open coding phase, any disagreements in coding decisions were resolved through discussion including the lead investigator (DC). These individual codes were subsequently grouped together into similar categories in order to generate themes pertaining to this topic (focused coding). Themes were identified and subsequently distilled and organized into overarching themes based on similarities and relationships between codes and themes.

### Trustworthiness

The study methodology is strengthened by having trained personnel with varied perspectives and experience who conducted the interviews and the analysis. Having multiple reviewers conduct the analysis also contributes to trustworthiness through bringing multiple lenses to bear on the data (triangulation).

## Results

We interviewed four pharmacists, from Calgary, Edmonton, Vancouver and Ottawa. Three of the pharmacists were male, and one was female. All four pharmacists were serving PWLEH who had diabetes. Three pharmacists were serving both PWLEH and the general population with a fee-for-service payment model. One pharmacist was funded directly by the government and solely targeted a low-income population.

**Figure 1:**
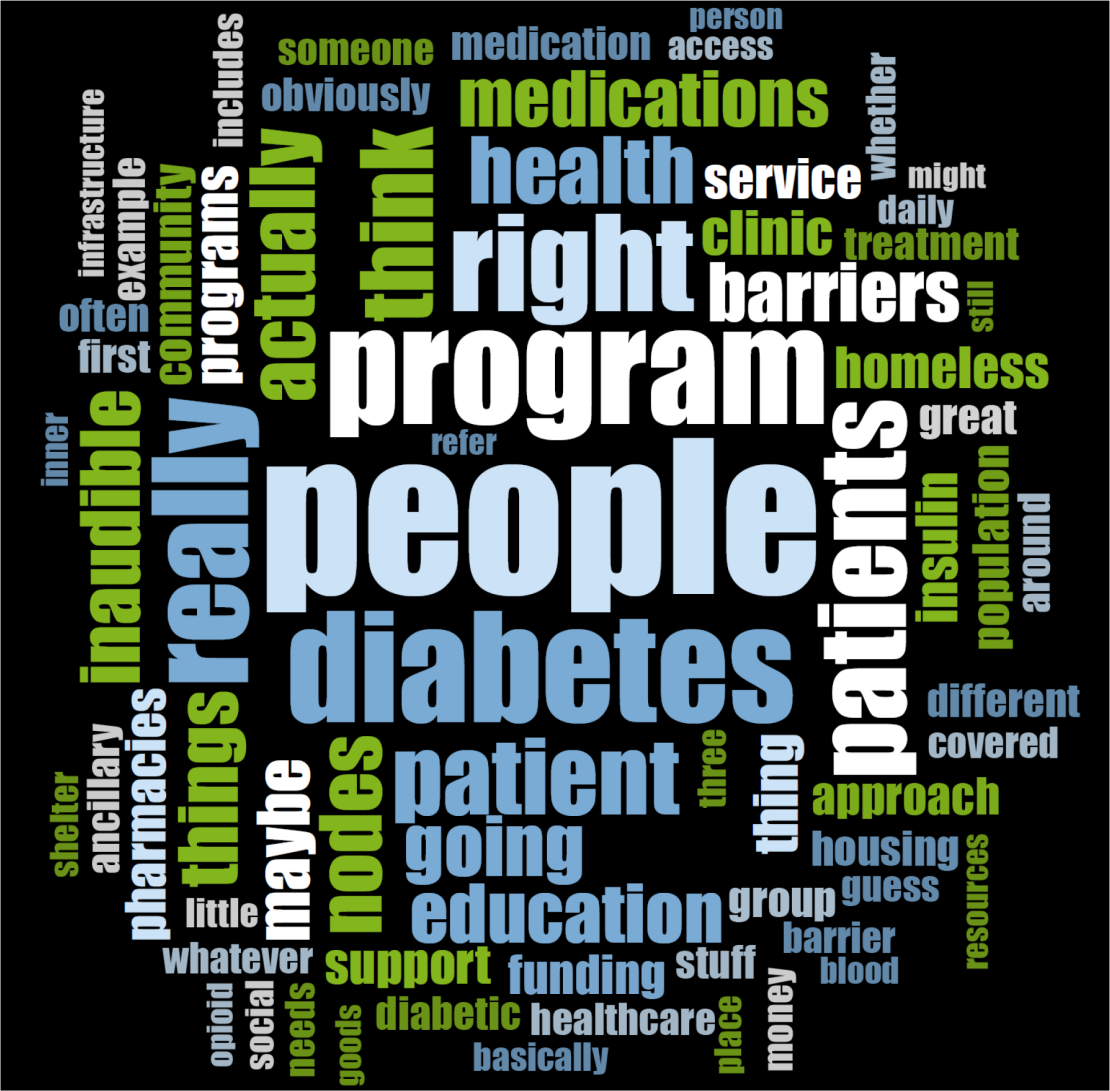
Word cloud illustrating most commonly used words.

The most common words mentioned in our transcripts are presented in a word cloud for illustrative purposes (**Figure 1**). Our thematic analysis yielded the following four themes:

### Development of diabetes care

Diabetes was not the primary disease focus for these pharmacists. The diabetes-specific programming was often developed after the pharmacist noted a need in the population. PWLEH are affected by high rates of mental health and substance use disorders, as well as infectious diseases (1, 15). By providing medical therapies for these conditions and their associated complications (e.g., opioid replacement, hepatitis C treatment), pharmacists explained that they began to see a gap in overall chronic disease management, and specifically as related to diabetes. The pharmacists were in a unique position to build on their existing relationships with their patients to provide care for their chronic conditions as well. A foundation of respect and a harm reduction approach aided this work:

*“So when you start to treat people for addiction, there are other comorbidities, they want to handle everything because they’ve never had somebody even shake their hand let alone even talk to them in the face because you know, there’s so much stigma among the homeless or low income”*. (Pharmacist 1).

### Tailored diabetes care for PWLEH

Pharmacists provided diabetes education that was tailored specifically to their patients’ needs and situations. In one city, individuals with diabetes are required to complete certified diabetes education to qualify for free self-monitoring supplies from the provincial health insurance plan. One pharmacist became a certified diabetes educator to help patients meet this requirement without having to attend generic mainstream group diabetes education, wherein much information and advice is given that does not apply to the reality of living on the streets or in a shelter. The diabetes education provided by the pharmacist was individualized:

*“I might talk about like hypoglycemia, right, and how to manage it, especially if they are on insulin and like if they have to skip meals, what to do … For some people like you know, if they are in addiction and they are drinking, I might go over diabetes and alcohol”.* (Pharmacist 2).

Pharmacists often see patients more frequently than other healthcare providers, especially those who visit the pharmacy for daily methadone, suboxone or managed opioid therapy. Some pharmacists even reported holding onto patients’ self-monitoring supplies for them, as theft of these supplies is common. These pharmacists would check patients’ blood glucose daily when they would come in for their daily dispensed medications. This frequent contact was used to complete medication reviews, educate patients, and adjust medications: *“So I have a certain patient that I see kind of chronically for things like insulin adjustments and just following, mainly insulin adjustments are people that I would see weekly”* (Pharmacist 2). These pharmacists also endorsed extended business hours and being open on weekends and evenings, to ensure adequate access for patients who might not be available during standard business hours.

### Involvement in other services caring for PWLEH

These pharmacists serving PWLEH were often uniquely integrated with other targeted social and health care services for PWLEH. The pharmacies were situated either within a community health center or within proximity to a community health clinic or shelter (e.g., one block) to ensure convenient access for PWLEH. One pharmacist described delivering medications to various shelters and addiction treatment facilities: *“we provide free delivery daily, or several times a day to every facility … we service about 40 different programs”* (Pharmacist 1).

These pharmacists provided services that went above and beyond the conventional services provided in community pharmacies. One pharmacy chain even funded supportive housing units and welcomed other agencies into their pharmacy:

*“If we are not housing immediately through that particular program, I’ll often, we have kind of partnered with a couple of agencies in the area, and we actually have a couple of their supportive housing workers come in meet with patients on site here.”* (Pharmacist 3).

Another pharmacy had a financial liaison who was able to help patients obtain medication coverage and identification documents. The pharmacists described having educated themselves about various social services for PWLEH to provide well-rounded care.

### Fiscal considerations

The main obstacle pharmacists faced was balancing the optimal medical care for their patients with diabetes and running a successful business. PWLEH often lose medications or have them stolen because they lack safe storage. Pharmacists’ solution to this problem was to dispense fewer days of medication at each fill. However, new billing rules in one province limited pharmacists on how frequently medications could be dispensed: *“The government left a massive hole in where a lot of things were being done on a weekly basis. What they basically said was, oh we are not going to pay you if you do that”* (Pharmacist 4). This provincial government also decreased funding for conducting comprehensive medication reviews, effectively disincentivizing pharmacists from undertaking this type of high-value care.

Other funding models may be more appropriate for those serving PWLEH. One of the pharmacies was only available to low-income disadvantaged individuals and was funded through the regional health authority, rather than through a private model, like the others:

*“[The] financial liaison worker … [has] to approve you to use our pharmacy which means that you would have to meet certain financial criteria in that you basically have to be low income”.* (Pharmacist 2).

One of the other private pharmacies was financially sustainable due to the large number of patients served, which offset the costs of the unique services like medication deliveries.

## Discussion

Some pharmacists who serve PWLEH in Canada have developed unique diabetes services due to an identified need in the population. These services required additional knowledge of the various social service programs and sometimes additional certification by pharmacists. Pharmacists have the advantage of frequent contact with patients, allowing time for medication reviews and education and the building of strong relationships with patients. At times, running a business and optimal patient care were noted to be at odds with one another.

Beggs et al. demonstrated that pharmacy-led chronic disease education improved medical knowledge for PWLEH (16). The pharmacists in our study identified a need for further diabetes education while providing care for other medical conditions like mental health and substance use disorders. They utilized their frequent contact with patients to provide individualized diabetes education including the use of insulin with food insecurity and substance use. In one setting, this individualized education allowed patients to receive coverage for blood glucose testing strips without needing to participate in traditional group education, which may be inapplicable to their needs. The pharmacists also developed connections to various social services, including housing support programs, so they could connect their patients with available non-medical services. This study supports Diabetes Canada’s recommendation that pharmacists be involved with diabetes education, as is evidenced through their unique role and advantages in educating PWLEH (8).

Studies by Johnson and Lowrie described a pharmacist-led outreach program in Glasgow, UK called PHOENIx in which they provided care at shelters, soup kitchens and even on the streets. Patients had positive interactions as it was found to be convenient to access care in these settings, without requiring appointments or transportation (17, 18). Although none of the pharmacists interviewed in this study provided care in shelters, outreach was provided by delivering medication to shelters and addiction programs. These pharmacies were also open late and located within the inner city, negating the need for transportation. Patients did not require appointments with their pharmacists and saw them frequently. Our study, in conjunction with prior literature, demonstrates how pharmacy locations, outreach, business hours, and flexible schedules can improve access to care for PWLEH (17, 18).

Bahrami et al. found pharmacy consultation improved A1C in PWLEH, validating the importance of pharmacists in diabetes management (13). Unfortunately, government funding for providing such consultative services was found in this study to be threatened in some jurisdictions. Defunding these services put the business interests at odds with providing optimal care. The pharmacists felt they could no longer provide the best care for PWLEH under the current reimbursement structure and remain solvent. Alternative funding models can be one means of reconciling these incentives. One pharmacy interviewed in this study was able to provide all medications necessary to their patients at no charge- including over-the-counter medications – through a special arrangement. Patients had to provide documentation to show that they were low-income in order to use this pharmacy, and it was directly funded by the regional health authority. These models are designed around the unique needs of PWLEH; frequent visits, medication reviews, and medication coverage allowed pharmacists to provide optimal care to improve diabetes outcomes without the competing financial demands of running a profitable business.

### Strengths and limitations

One strength of this study was the inclusion of pharmacists from three provinces, each having its own unique billing and funding structure, making our findings relevant to multiple locations. These pharmacists had unique backgrounds and connections within their communities, allowing for a broad range of programming. Limitations of the study include the small number of participants, and the fact that these pharmacists were chosen and approached for participation, which has an impact on the representativeness of the data. This study also only focused on the perspective of the pharmacists and did not include the perspective of PWLEH. However, we continue to engage with PWLEH in parallel research endeavours to ensure their voices are heard and amplified (5, 7, 19). Future research may focus on the perspective of health authorities and system payers, specifically regarding how various funding models affect the experience of PWLEH and diabetes at pharmacies and their impact on clinical outcomes.

## Study implications

Pharmacists have a vital role in helping to manage diabetes in PWLEH. Pharmacists have the benefit of frequent contact with patients, allowing continued education and medication adjustments. The pharmacists in this study developed connections with various social programs in their community to provide holistic care. Pharmacists can continue to serve their patients by creating novel programming in order to address the particular challenges faced by PWLEH. However, it is important that government funding is structured in a manner to support the unique needs of pharmacists that care for this group of patients to enable them to provide optimal care for PWLEH.

## Data availability statement

The datasets presented in this article are not readily available because this is not permitted by our ethics approval. Given the qualitative nature of our data and the small sample size, anonymization could never be guaranteed on a dataset of this nature. Requests to access the datasets should be directed to David J.T. Campbell, dcampbel@ucalgary.ca.

## Ethics statement

The studies involving human participants were reviewed and approved by Research Ethics Boards of the University of Calgary and Unity Health Toronto/St. Michael’s Hospital. The patients/participants provided their written informed consent to participate in this study.

## Author contributions

DC conceptualized and developed the methodology. RC and DC conducted the interviews. RC, EG, DC completed the initial coding of the interviews. BM and DC completed the focused coding of the interviews and wrote the original manuscript. All authors contributed to the article and approved the submitted version.
