# Supplementary material for: Pharmacists’ role in diabetes management for persons with lived experience of homelessness in Canada: A qualitative study
Source: Front Clin Diabetes Healthc. 2022 Dec 22;3:1087751. doi: 10.3389/fcdhc.2022.1087751 (PMC10012085; doi:10.3389/fcdhc.2022.1087751)
Supplement: Supplementary file 1 [file Table_1.docx]

| **Appendix A: SRQR Checklist** | | | | |
| --- | --- | --- | --- | --- |
| **Number** | **Topic** | **Page** | **Line** | **Excerpt** |
| S1 | Title | 1 | 1-2 | Pharmacists' Role in Diabetes Management for Persons with Lived Experience of Homelessness in Canada: A qualitative study |
| S2 | Abstract | 2 |  |  |
| S3 | Problem Formulation | 3 | 102-115 | Information on how pharmacists design and tailor their practice to achieve these improved outcomes for PWLEH is limited. |
| S4 | Purpose or Research Question | 3 | 117-119 | Our objective was to describe the approaches of four pharmacists across Canada who work closely with PWLEH. A prior publication by our team discussed the general model of care (11) but here we discuss the details of how pharmacists can provide diabetes care for PWLEH. |
| S5 | Approach and Paradigm | 3 | 123 | We conducted a multi-site qualitative descriptive study using the constructivist paradigm. |
| S6 | Researcher Characteristics | 4 | 139-141 | Data were collected using semi-structured in-person qualitative interviews, conducted by research personnel (RBC and DJTC) who are experienced in qualitative data collection and analysis, and who have expertise in homelessness and diabetes care for PWLEH, respectively. |
| S7 | Context | 3 | 123-127 | We interviewed providers from programs that were tailored to address the needs of people experiencing homelessness with diabetes in four large Canadian urban centres (Calgary, Edmonton, Vancouver and Ottawa). The interviews took place between July 2018 and February 2019. |
| S8 | Sampling Strategy | 4 | 132-136 | We recruited participants who had at least 3 years of providing direct clinical care to those with diabetes and homelessness. We sought pharmacists who worked in inner-city settings serving PWLEH and who had an explicit focus or program for these populations. These pharmacists were recruited through internet searches and snowball sampling of health agencies serving PWLEH in these cities. |
| S9 | Ethical Issues | 3 | 127-128 | This study was approved by the Research Ethics Boards of the University of Calgary and Unity Health Toronto/St. Michael’s Hospital… Written informed consent was obtained from each participant. |
| S10 | Data collection methods | 4 | 139-147 |  |
| S11 | Data collection instruments | 4 | 142-146 | Pre-defined interview guides were created (Appendix A); these were iterative and after each interview, the study team members reconsidered the interview guide, adding additional questions to probe into areas that were revealed in prior interviews. Interviews ranged from 20-90 minutes in duration. |
| S12 | Units of study | 4 | 160-164 | We interviewed four pharmacists, from Calgary, Edmonton, Vancouver and Ottawa. Three of the pharmacists were male, and one was female. All four pharmacists were serving PWLEH who had diabetes. Three pharmacists were serving both PWLEH and the general population with a fee-for-service payment model. One pharmacist was funded directly by the government and solely targeted a low-income population. |
| S13 | Data processing | 4 | 146-147 | Interviews were digitally recorded, and recordings were subsequently transcribed verbatim by a trained professional. |
| S14 | Data analysis | 4 | 149-157 |  |
| S15 | Techniques to enhance trustworthiness | 4 | 159-163 | The study methodology is strengthened by having trained personnel with varied perspectives and experience who conducted the interviews and the analysis. Having multiple reviewers conduct the analysis also contributes to trustworthiness through bringing multiple lenses to bear on the data (triangulation). |
| S16 | Synthesis and interpretation | 5-6 | 166-248 | Results section |
| S17 | Links to empirical data | 5-6 | 166-248 | Quotes throughout |
| S18 | Integration, implications, transferability | 6-8 | 250-312 | Discussion section |
| S19 | Limitations | 7 | 299-305 | Limitations of the study include the small number of participants, and the fact that these pharmacists were chosen and approached for participation, which has an impact on the representativeness of the data. This study also only focused on the perspective of the pharmacists and did not include the perspective of PWLEH. However, we continue to engage with PWLEH in parallel research endeavours to ensure their voices are heard and amplified (5,7,16). |
| S20 | Conflicts of Interest | 8 | 319-321 | The funders had no role in the study conduct, analysis, or interpretation of results. The authors report no conflicts of interest. |
| S21 | Funding | 8 | 315-317 | Funding for this study was provided by the Wenzel Family Cardiometabolic Fund, the O’Brien Institute Program for Research with Vulnerable Populations, and an Alberta Innovates Clinician Fellowship awarded to Dr. Campbell. |
