# Supplementary material for: Pharmacists’ role in diabetes management for persons with lived experience of homelessness in Canada: A qualitative study
Source: Front Clin Diabetes Healthc. 2022 Dec 22;3:1087751. doi: 10.3389/fcdhc.2022.1087751 (PMC10012085; doi:10.3389/fcdhc.2022.1087751)
Supplement: Supplementary file 2 [file Table_2.docx]

**Appendix B: Interview Guide**

- Is your practice tailored to the experience of those experiencing homelessness?
  - Time/day offered? Always same time? Location? Who staffs? How advertised?
- What does your program do?
  - What services are offered (group classes, individual appointments, education, outreach, etc…
- Does your program/service offer any diabetes-specific care?
- How are the specific barriers your patients face considered in your practice?
  - Housing instability
  - Medication & testing supply access/storage/administration
  - Addictions
  - Mental health
  - Food insecurity
- What unique challenges does this population face in self-management of chronic conditions?
  - What unique challenges do you think those with diabetes face?
- Do you facilitate patients’ access to ancillary health services, like a podiatrist, optometrist, or dietician?
- Do you facilitate patients’ access to government and community supports, such as the food bank, social workers, etc?
- What are the strengths of your program? What works well?
- Do you think this program has been able to achieve improved health outcomes for your target population? What outcomes?
  - Improved glycemic control
  - Reduced hospitalizations
  - Fewer diabetes-related complications
- Do you think this program has been able to achieve improved psycho-social outcomes for your target population? What outcomes?
  - Improved quality of life
  - Improved mental health
  - Increased stability
  - Greater ownership and sense of control over disease
  - Others?
- Are you aware of resources to which you might refer clients with diabetes? Any that are tailored to this population (poor or marginalized populations who may face barriers to accessing care, self-management, and allied health services)?
